# Oleanolic acid enhanced the anticancer effect of fluorouracil by regulating Ca^2+^ levels in hepatocellular carcinoma cells

**DOI:** 10.1590/1414-431X2025e14590

**Published:** 2025-04-14

**Authors:** Jing Dong, Yin Gao, Penghui Li, Ping Chen, Yanxin Lv, Yanan Liu, Song Zhang, Minglong Zhang, Yu Wang

**Affiliations:** 1 Department of Medical Cell Biology, Qiqihar Medical University, Heilongjiang, China; 2 Department of Nursing Laboratory, Qiqihar Medical University, Heilongjiang, China; 3 Department of Electronics, Qiqihar Medical University, Heilongjiang, China; 4 Department of Medical Genetics, Qiqihar Medical University, Heilongjiang, China

**Keywords:** Oleanolic acid, Fluorouracil, Hepatocellular carcinoma, Calcium ion, Apoptosis

## Abstract

Oleanolic acid (OA) is recognized for its anticancer properties, which are similar to those of conventional chemotherapeutic agents used in clinical practice. However, its role in modulating the sensitivity of cancer cells to fluorouracil (5FU) has not yet been documented. This study aimed to examine the effects of OA and 5FU co-administration on hepatocellular carcinoma (HCC) and uncover the mechanisms involved. In this study, the efficacy of combination therapy with OA and 5FU in treating HCC was evaluated using the MTT cell proliferation assay, plate clone formation assay, Hoechst 33342 staining, western blot assay, and Ca^2+^ fluorescence probe. The results demonstrated that compared with the use of OA or 5FU alone, OA and 5FU combination therapy significantly inhibited the proliferation of HEPG2 cells and enhanced cell apoptosis and Ca^2+^ levels in HCC. Additionally, the inhibitory effect of OA and 5FU combination therapy on cell proliferation and apoptosis was partially reversed by the calcium channel blocker 2-aminoethyldiphenyl borate (2-APB). In summary, these findings indicated that synergistic treatment with OA and 5FU can enhance cell apoptosis, inhibit cell proliferation, and regulate Ca^2+^ signaling in HCC, providing new guidance for the clinical treatment of HCC.

## Introduction

Hepatocellular carcinoma (HCC) is the most common primary malignant tumor of the liver and is usually caused by chronic liver disease or cirrhosis (1). Liver cancer is the leading cause of cancer-related deaths globally (2). Surgical resection is currently the preferred treatment for early HCC patients (3). However, surgery is only suitable for a small number of patients (4). It is usually limited to cases in which the tumor is small and confined to the liver (5). Targeted therapy also plays an important role in HCC (6). Liu et al. (7) found that CANT1 is significantly overexpressed in hepatocellular carcinoma tissues, and its high expression indicates poor prognosis for patients. Zhang et al. (8) found that systemic treatment with PD-1/PD-L1 inhibitors can effectively treat HCC, prolonging the survival of HCC patients. Despite the continuous improvement in treatment methods for HCC in recent years and the extension of patient survival, the overall prognosis is still not ideal (9). Therefore, exploring new therapeutic targets and combination therapies could further improve treatment efficacy and prognosis of patients with HCC.

Fluorouracil (5FU) is a commonly used antimetabolic chemotherapeutic drug that is a pyrimidine analogue (10) extensively used in treating various cancers, including colorectal (11) and gastric cancer (12). 5FU was first introduced in clinical practice in 1957 and remains a key drug in diverse chemotherapy regimens for malignant tumors to this day (13). 5FU inhibits HCC proliferation by interfering with DNA and RNA synthesis in the cancer cells (14). However, the specific mechanism of action of 5FU treatment in HCC is currently obscure and requires further investigation.

Oleanolic acid (OA) is a naturally occurring pentacyclic triterpenoid extensively distributed in diverse plants (15). OA has anti-inflammatory, antioxidant, antitumor, antiviral, and liver protective effects (16). Alqrad et al. (17) found that OA has significant anti-inflammatory effects that can inhibit the release of inflammatory factors and alleviate inflammatory reactions. Abulaiti et al. (18) found that OA exhibited broad-spectrum antitumor activity and inhibited the malignant progression of cancer cells. He et al. (19) found that OA had a significant inhibitory effect on the development of breast tumors in mice. Li et al. (20) found that OA has a protective effect on the liver, alleviating chemical liver damage, promoting liver cell regeneration, and preventing the occurrence of liver fibrosis to some extent. Calcium ions (Ca^2+^) play crucial roles in regulating cell proliferation and apoptosis. Tumor cells maintain high levels of cell proliferation and survival by altering the influx and efflux of calcium ions and their storage in organelles (21).

Herein, we investigated the effects of the OA/5FU combination therapy on cancer and its potential regulatory mechanisms on calcium levels in cancer cells. This study is the first to explore the potential of OA/5FU combination therapy for HCC and to provide new directions for anti-cancer strategies.

## Material and Methods

### Cell culture and reagents

The human liver cancer cell line HEPG2 was purchased from the Cell Bank of the Institute of Experimental Animal Science, CAMS & PUMC (China). Cells were cultured in Dulbecco's modified Eagle's medium (DMEM) (Gibco, USA) containing 1% streptomycin (100 mg/L), penicillin (100 U/L), and 10% fetal bovine serum (FBS). Cells were incubated in a 5% CO_2_ incubator at 37°C. The main reagents used in this study are as follows: OA (Beijing Soleibao Technology Co., Ltd., SO8030, China); 5FU (Beijing Soleibao Technology Co., Ltd., IF0170); Rainbow 180 broad spectrum protein marker (Beijing Soleibao Technology Co., Ltd., PR1910); 2-Aminoethyl diphenyl borate (2-APB) (MedChemexpress Biotech, HY-W009724, USA); Mag-Fluo-4 calcium ion indicator (Thermo Fisher Scientific, USA, M14206); Rhod-2AM calcium ion fluorescent probe (Abcam, UK, ab142780); D-Hanks’ solution (PYG0079, Wuhan Doctoral Biotechnology Co., Ltd., China); MTT Cell Proliferation and Cytotoxicity Detection Kit (Wuhan Boside Bioengineering Co., Ltd., AR1156, China); Hoechst 33342 staining solution (Wuhan Boside Bioengineering Co., Ltd., AR0039, China); Polyoxymethylene tissue fixation solution (Wuhan Boside Bioengineering Co., Ltd., AR1068).

### Cell viability evaluation

Liver cancer HEPG2 cells were inoculated onto a 96-well plate at a density of 5000 cells per well. After culturing until the cells adhered to the wall and reached 80% confluence, different concentrations of the drugs were added to each well to establish a control group. Cultivation was continued in an incubator for 24, 48, or 72 h. Then, 10-20 µL of MTT solution was added to each well, with a final concentration of 0.5 mg/mL, the plate was incubated for 4 h, and 200 µL of DMSO was added. The plate was gently shaken on a shaker for 10 min to ensure complete dissolution of the cells. The absorbance of each well was measured at 490 nm using an enzyme-linked immunosorbent assay (ELISA) reader. The cell survival and proliferation rates were computed based on the absorbance value relative to the untreated control group.

### Colony formation assay

Hepatocellular carcinoma cells fused, grew to approximately 90% confluence, and were digested with trypsin to form a single-cell suspension. Cell counting was performed by seeding cells at a density of 200 cells/well. Incubation was done in a 6-well plate for 14 days at 37°C in a 5% CO_2_ incubator. The culture medium was replaced every two days to ensure that the cells received sufficient nutrition. After clone formation, the culture medium was discarded, and the cells were rinsed with PBS and fixed with 4% paraformaldehyde for 15 min. The fixative was discarded. Giemsa staining solution was added for 30 min until the clone was clearly visible. The plate was gently rinsed with tap water and was inverted onto absorbent paper and air-dried.

### Hoechst 33342 staining

Before processing the HEPG2 cells, a glass slide was added to the well plate. After routine digestion, the cells were cultured in a well plate. When the cells fused and reached over 80% confluence, they were washed with PBS and then fixed with 4% paraformaldehyde for 15 min. The cells were washed thrice with PBS after fixation for 5 min each. An appropriate amount of the Hoechst 33342 working solution was added to the cells to ensure complete coverage. They were incubated in a 37°C incubator for 30 min. After staining, the cells were gently washed with PBS and protected from light. The cells were observed under a fluorescence or confocal microscope.

### Western blot analysis

After collection, cultured cells were washed with cold PBS. RIPA buffer and protease inhibitor mixture were added, incubated on ice for 30 min, and intermittently vortexed and shaken during this period. After cell lysis, centrifugation was done at 4°C and 13,400 *g* for 20 min, and the protein was collected from the supernatant. The total protein concentration in the samples was quantified using the BCA method. SDS-PAGE gel was prepared in advance. The sample was mixed with 2× SDS loading buffer and heated at 95°C for 5 min to denature the protein. A constant voltage of 80-120 V was applied during electrophoresis. After electrophoresis was completed, the separation gel was cut off, and the PVDF membrane was placed in a transfer fixture for semi-dry transfer. The PVDF membrane was transferred to a solution containing 5% non-fat milk powder and sealed at room temperature for 1 h. The primary antibody was added and incubated overnight on a shaker at 4°C. The next day, the membrane was washed thrice with TBST solution for 10 min each time. Secondary antibody solution was added and incubated for 1 h. The membrane was washed thrice with TBST solution, and chemiluminescence detection of the bands was performed for 10 min each time.

### Detection of the cytoplasmic and mitochondrial Ca^2+^ levels

HEPG2 cells in 24-well plates were treated with different drugs for 72 h. After removing the supernatant, the cells were washed with D-Hanks' solution, which was discarded. The mitochondrial Ca^2+^ fluorescent probe Rhod-2AM (Abcam) and cytoplasmic Ca^2+^ fluorescent probe Mag-fluo-4AM (Thermo Fisher Scientific) (500 µL) were added into each well, respectively. The cells were incubated with the fluorescent probe at 37°C in the dark for 30 min. After washing the cells with D-Hanks' solution and discarding the solution, cytoplasmic and mitochondrial Ca^2+^ levels were observed under a fluorescence microscope (Olympus, Japan).

### Flow cytometry

HEPG2 cells (1×10^5^ cells/well) were seeded onto 6-well culture plates. After 24 h, cells were treated with the drug for 72 h. According to the manufacturer's instructions, cells were collected and washed twice with pre-cooled D-Hank's buffer. After washing, 500 µL of fluorescence dye solution (2.5 µM Rhod-2AM or 5 µM Mag-fluo-4AM) was added and incubated for 30 min at room temperature away from light. HEPG2 cells were harvested and washed with pre-cooled D-Hank's buffer. The cells were then analyzed by flow cytometry (Becton Dickinson, USA).

### Statistical analysis

Data analyses were conducted using SPSS (version 26.0, IBM, USA) and GraphPad Prism (version 9.0, USA). All observations were verified in at least three independent experiments. The mean differences between the control and treatment groups were compared using one- or two-way ANOVAs, followed by Tukey's *post hoc* multiple comparison test. Data are reported as means±SD. P<0.05 indicated a significant difference.

## Results

### OA combined with 5FU inhibited the proliferation ability of HCC cells

MTT assay revealed that OA (20-150 μM) and 5-FU (2.5-80 μM) had a significant inhibitory effect on the growth of human liver cancer HEPG2 cells. However, when the concentration of 5-FU reached 40 μM, further increases in 5-FU concentration did not result in a significant change in the inhibition rate. When OA and 5-FU were combined at a concentration ratio of 5:1 to treat HEPG2 cells, the combination exhibited a synergistic inhibitory effect on cell growth compared to the monotherapy group (P<0.05). Analysis using Calcusyn 2.0 software indicated that the combination index (CI) was less than 1 across most concentration ranges. Based on the effects of the drug combination therapy on cell proliferation, we then selected the 100 μM OA and 20 μM 5-FU combination therapy as working concentrations for subsequent research (Supplementary Figure S1).

**Figure 1:**
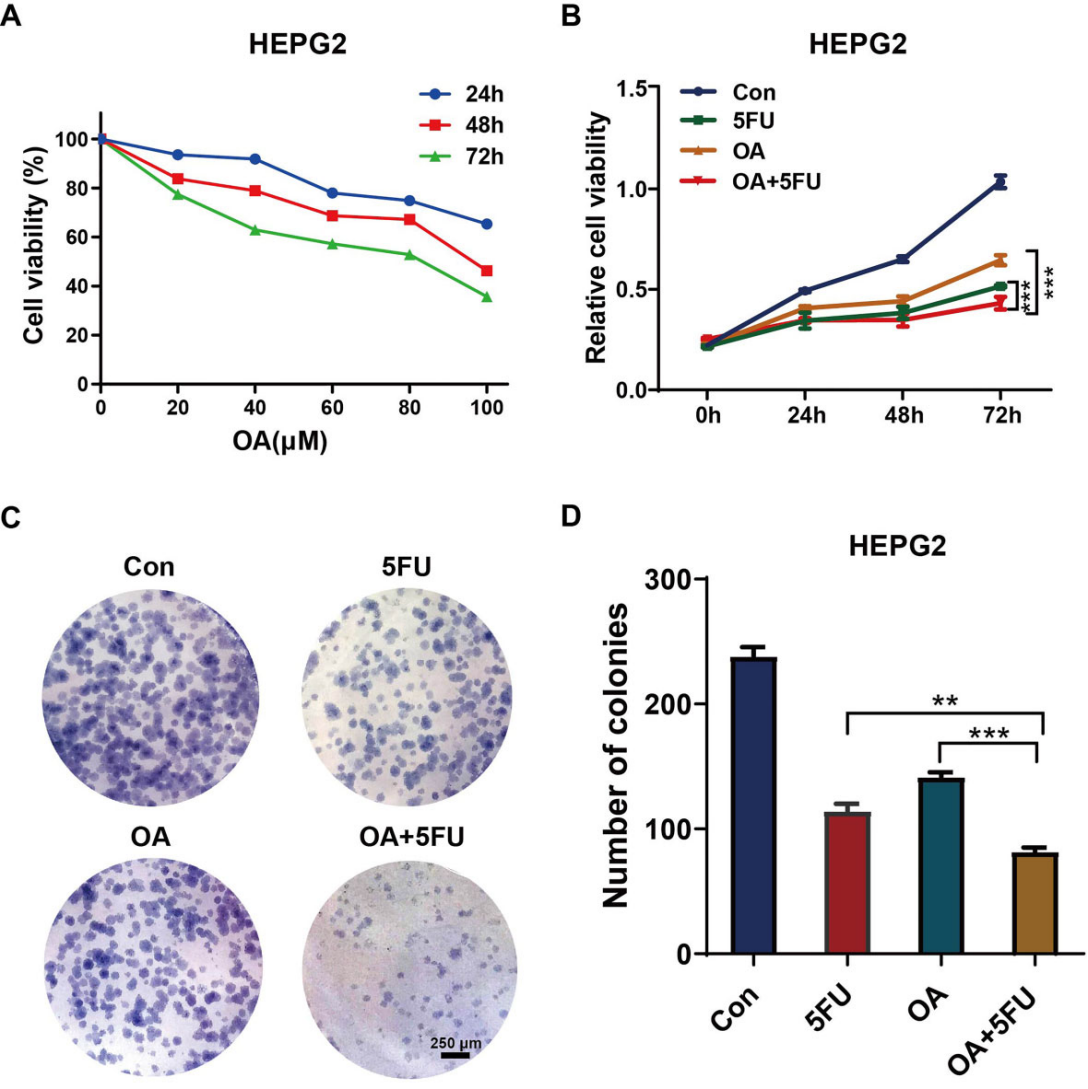
Oleanolic acid (OA) and fluorouracil (5FU) co-treatment inhibited the proliferation ability of hepatocellular carcinoma (HCC) cells. **A**, The effect of different concentrations and time-point of OA treatment on cells was determined by MTT. **B**, The influence of OA and 5FU co-treatment on the cell proliferation ability was determined by the MTT assay. **C** and **D**, The influence of 5FU or OA alone and OA/5FU co-treatment on the colony formation ability was determined by the colony formation assay (scale bar, 250 μm). Data are reported as means±SD from three independent experiments. **P<0.01; ***P<0.001; ANOVA. Con: control. Co-treatment: 100 μM OA and 20 μM 5-FU.

Further cell proliferation experiments revealed that, compared to the control group, OA or 5FU alone significantly inhibited the activity of HCC, and the OA/5FU combination therapy had a more significant effect (Figure 1A and B). To further verify the effect of OA combined with 5FU on the proliferation of HCC cells, plate cloning experiments revealed that OA combined with 5FU significantly reduced the HEPG2 cell count (Figure 1C and D). The results demonstrated that OA combined with 5FU significantly inhibited HCC cell proliferation.

### OA combined with 5FU induced apoptosis in HCC cells

**Figure 2:**
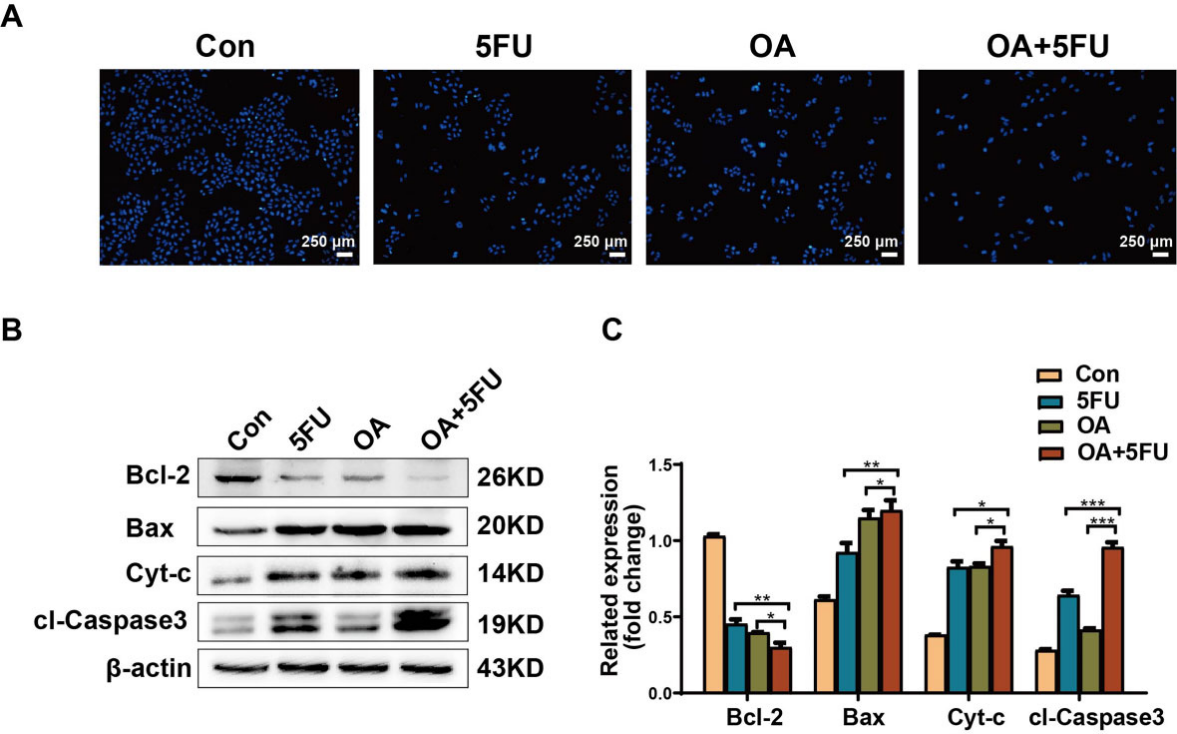
Effects of oleanolic acid/fluorouracil (OA/5FU) co-treatment on apoptosis of HEPG2 cells. **A**, The influence of 5FU, OA, and OA/5FU co-treatment on the apoptosis of HEPG2 cells was determined by Hoechst 33342 staining (scale bar, 250 μm). **B** and **C**, The promoting effect of 5FU, OA, and OA/5FU co-treatment on pro-apoptotic proteins in liver cancer cells was assessed by western blot. Data are reported as means±SD from three independent experiments. *P<0.05, **P<0.01, ***P<0.001; ANOVA. Con: control.

The Hoechst 33342 staining assay demonstrated that the OA/5FU combined therapy had a stronger ability to induce apoptosis in HCC cells than OA or 5FU alone (Figure 2A). The expression of apoptosis-related proteins was further validated using western blotting. The results demonstrated that compared with OA or 5FU treatment alone, OA/5FU combined treatment significantly downregulated the anti-apoptotic protein Bcl-2. The pro-apoptotic proteins Bax, Cyt-c, and cl-Caspase-3 were significantly upregulated (Figure 2B and C). These results indicated that OA/5FU combination therapy can regulate the expression of apoptosis-related proteins and induce cancer cell apoptosis.

### OA combined with 5FU enhanced Ca^2+^ levels in HCC cells

**Figure 3:**
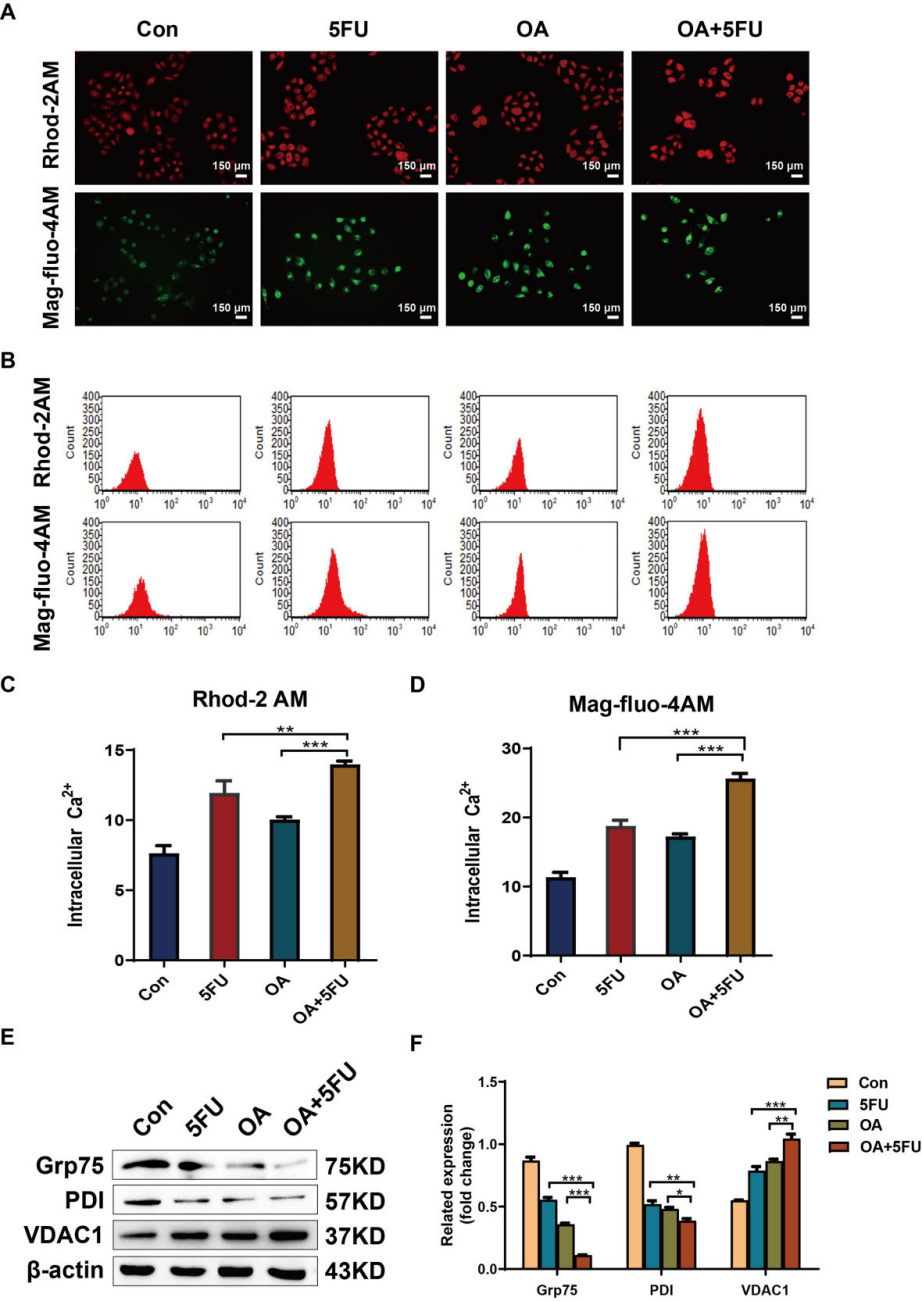
Effects of fluorouracil (5FU), oleanolic acid (OA), and OA/5FU co-treatment on Ca^2+^ of HEPG2 cells. **A**, Influence of different drugs on the cytoplasmic and mitochondrial Ca^2+^ level after the treatment of HEPG2 cells with the Ca^2+^ fluorescent probes Rhod-2AM and Mag-fluo-4AM (scale bar, 150 μm). **B**-**D**, After Ca^2+^ fluorescent probes Rhod-2AM and Mag-fluo-4AM were used to treat HEPG2 cells, flow cytometry was used to detect the effects of different drugs on cytoplasm and mitochondrial Ca^2+^ levels. **E** and **F**, Effects of 5FU, OA, and OA/5FU co-treatment on calcium channel-related proteins by western blot. Data are reported as means±SD from three independent experiments. *P<0.05; **P<0.01; ***P<0.001; ANOVA. Con: control.

To investigate the effect of OA/5FU combination therapy on Ca^2+^ levels in HCC cells, the mitochondrial Ca^2+^ fluorescent probe Rhod-2AM and cytoplasmic Ca^2+^ fluorescent probe Mag-flou-4AM were added to cancer cells. The results demonstrated that, compared with the application of OA or 5FU alone, the combination of the two drugs significantly enhanced the fluorescence intensity of HCC cells (Figure 3A). Flow cytometry analysis demonstrated that compared with the control group cells and the groups treated with 5FU and OA alone, the fluorescence intensity of the Ca^2+^ signal in HCC cells treated with the OA/5FU combination therapy was significantly increased (Figure 3B-D). Western blot analysis further detected the expression of Ca^2+^ channel-related proteins (Figure 3E and F). The results demonstrated that compared with the control group and the 5FU and OA treatment groups alone, the OA/5FU combination treatment group had downregulated Grp75 and PDI, whereas VDAC1 was significantly upregulated. These results indicated that OA/5FU combination therapy can synergistically enhance Ca^2+^ levels in cancer cells.

### 2-APB reversed the Ca^2+^ enhancement induced by OA/5FU combination therapy in HEPG2 cells

**Figure 4:**
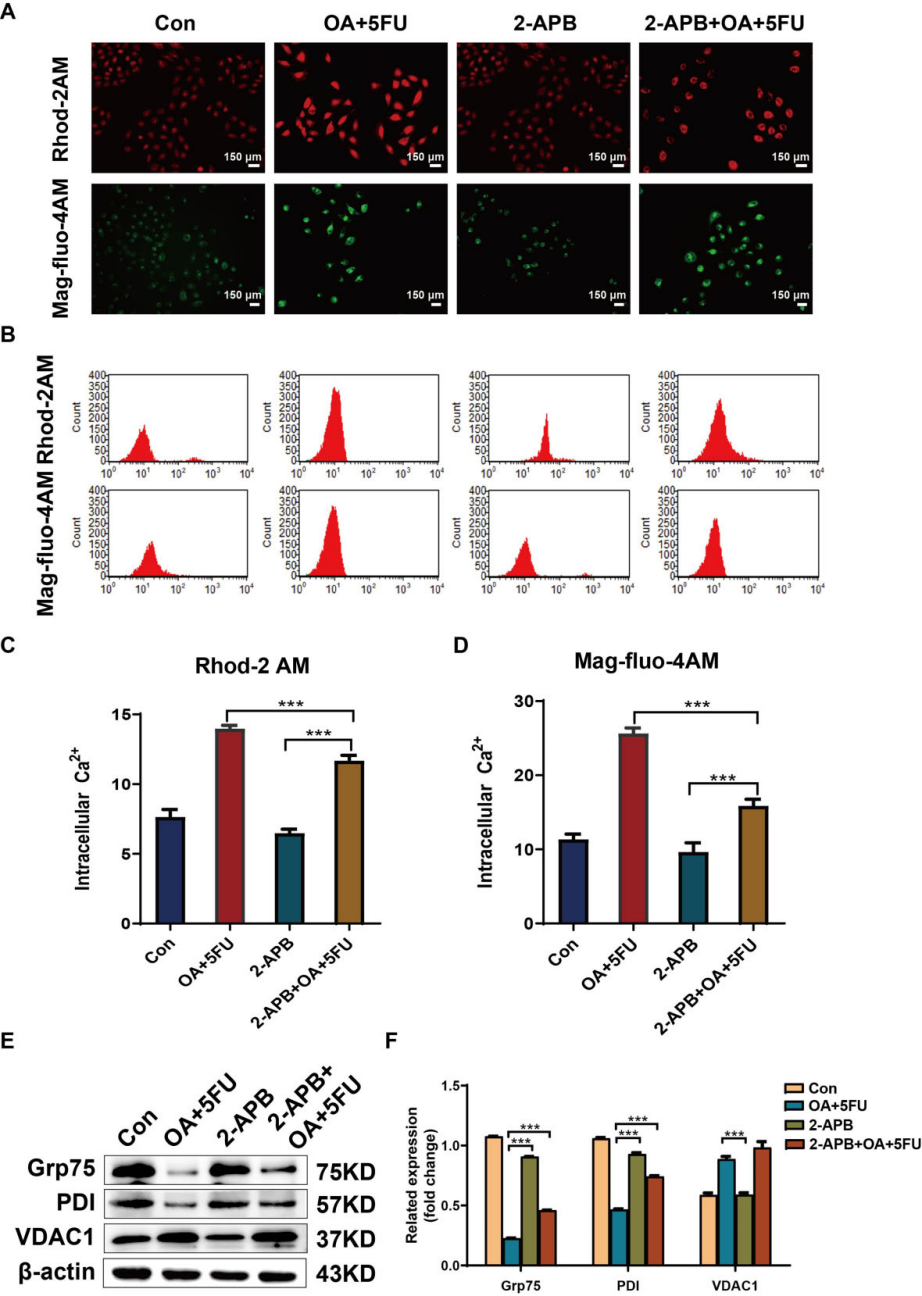
Effects of oleanolic acid/fluorouracil (OA/5FU) co-treatment on Ca^2+^ of HEPG2 cells under the action of 2-APB. **A**, The influence of different drugs on cytoplasmic and mitochondrial Ca^2+^ levels after the treatment of HEPG2 cells with the Ca^2+^ fluorescent probes Rhod-2AM and Mag-fluo-4AM with application of 2-APB (scale bar, 150 μm). **B**-**D**, Ca^2+^ fluorescent probes Rhod-2AM and Mag-fluo-4AM were used to treat HEPG2 cells, and flow cytometry was used to detect the effects of different drugs on cytoplasm and mitochondrial Ca^2+^ levels under the action of 2-APB. **E** and **F**, The effect of OA/5FU co-treatment on calcium channel-related proteins under the action of 2-APB was determined by western blot. Data are reported as means±SD of three independent experiments. ***P<0.001, ANOVA. Con: control.

2-APB was used as a Ca^2+^ channel inhibitor to further evaluate its application efficiency. Immunofluorescence and flow cytometry experiments demonstrated that, compared to the OA/5FU combination therapy group, 2-APB significantly reduced the fluorescence intensity of HCC cells. After co-treatment with 2-APB+OA/5FU, this phenomenon was reversed in HCC cells (Figure 4A-D). Western blotting was performed to detect the effects on calcium channel-related protein expression. The results demonstrated that the addition of 2-APB reversed the weakening of Grp75 and PDI expression in OA/5FU co-treated cells. The addition of 2-APB reduced the expression level of VDAC1. However, co-treatment of cells with 2-APB and OA/5FU reversed the expression pattern of 2-APB (Figure 4E and F). These results indicated that 2-APB can inhibit the Ca^2+^ efflux caused by combination therapy.

### 2-APB reversed the proliferation ability of HEPG2 cells inhibited by OA/5FU combined treatment

**Figure 5:**
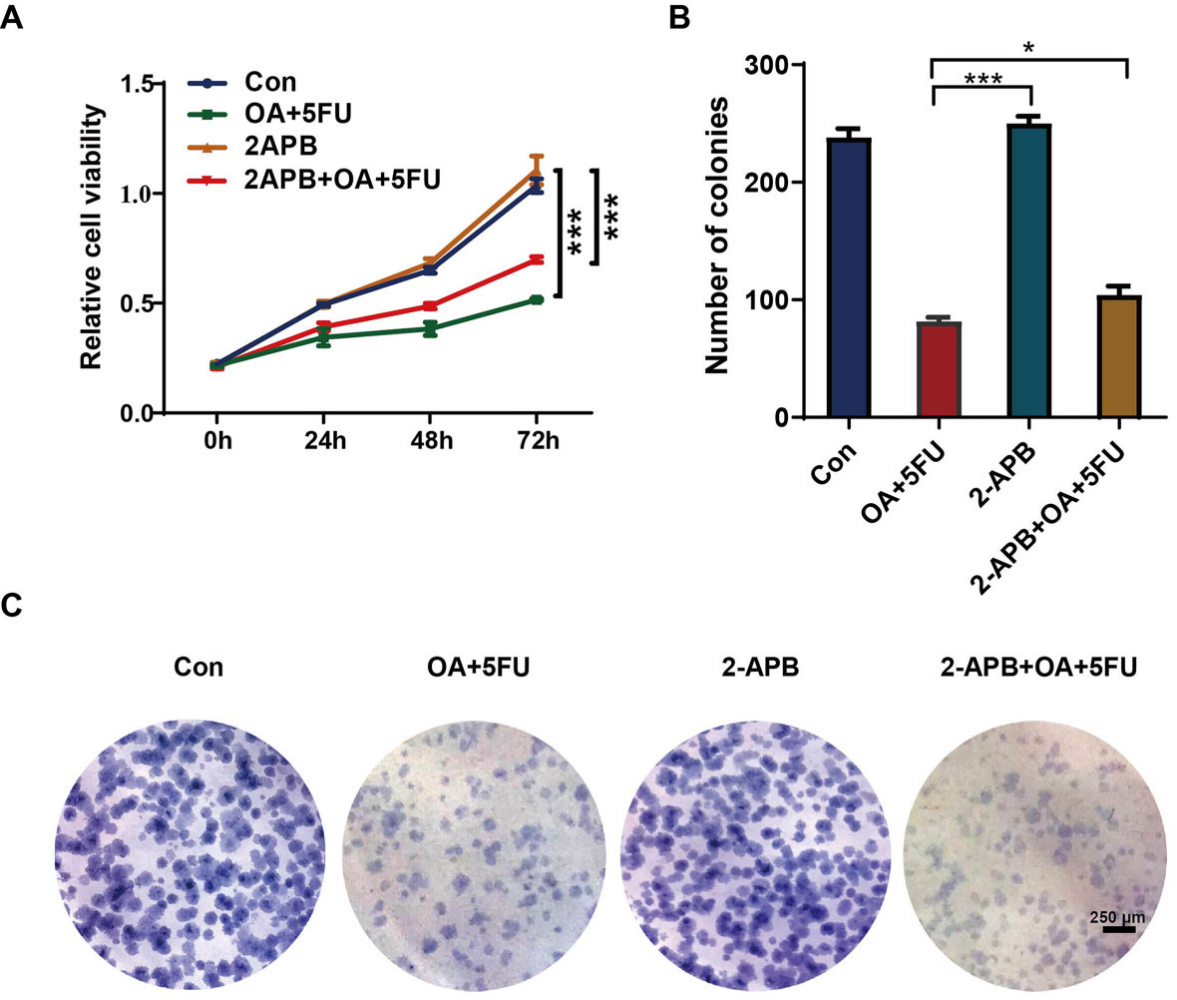
Effects of oleanolic acid/fluorouracil (OA/5FU) co-treatment on survival rate and proliferation rate under the action of 2-APB. **A**, The influence of OA/5FU co-treatment on cell proliferation under the action of 2-APB was determined by the MTT assay. **B** and **C**, The influence of OA/5FU co-treatment under 2-APB on the colony formation ability was determined by the colony formation assay (scale bar, 250 μm). Data are reported as means±SD from three independent experiments. *P<0.05; ***P<0.001; ANOVA.

We investigated whether the calcium channel inhibitor 2-APB affects the proliferative ability of HCC cells. MTT experiments demonstrated that compared with OA/5FU combination therapy, the application of 2-APB significantly promoted cell proliferation, while the combination therapy of 2-APB+OA/5FU weakened cell proliferation (Figure 5A). Further experiments on plate clone formation demonstrated that, compared with the OA/5FU combination therapy, the application of 2-APB significantly promoted the ability of cells to form plate clones. However, combination therapy with 2-APB+OA/5FU weakened the ability of the cells to form plate clones (Figure 5B and C). These results indicated that 2-APB can partially reverse the inhibitory effect of OA/5FU combination therapy on cell proliferation.

### 2-APB inhibited apoptosis of HEPG2 cells induced by OA/5FU combination therapy

**Figure 6:**
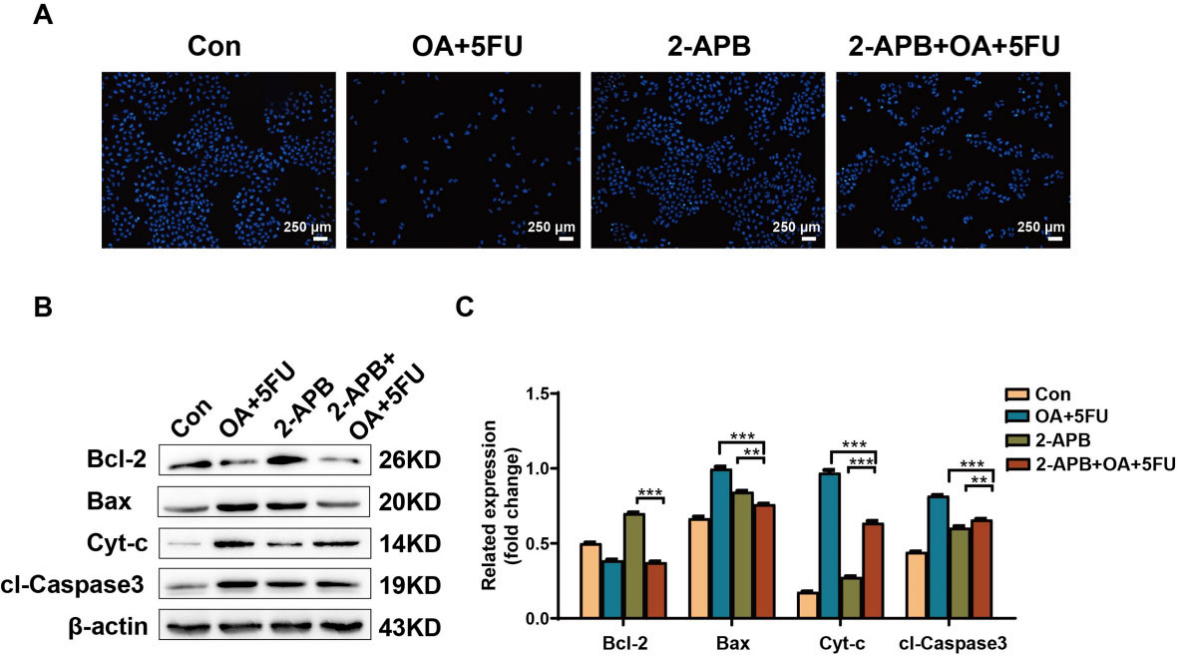
Effects of oleanolic acid/fluorouracil (OA/5FU) co-treatment on apoptosis of HEPG2 cells under the action of 2-APB. **A**, The influence of OA/5FU co-treatment on the apoptosis of HEPG2 cells under the action of 2-APB was determined by Hoechst 33342 staining (scale bar, 250 μm). **B** and **C**, The effect of OA/5FU co-treatment on the apoptosis of liver cancer cells under the action of 2-APB was determined by western blot. Data are reported as means±SD of three independent experiments. **P<0.01; ***P<0.001; ANOVA.

Hoechst 33342 staining demonstrated that 2-APB application partially reversed the apoptosis promoting effect of the OA/5FU combination therapy (Figure 6A). Western blotting was performed to detect the expression of apoptosis-related proteins. The results also demonstrated that the effect of OA/5FU combination therapy on apoptosis-related protein expression was partially reversed by 2-APB application. After the application of 2-APB, the pro-apoptotic proteins Bax, Cyt-c, and cl-Caspase-3 were significantly reduced, whereas the anti-apoptotic protein Bcl-2 was significantly enhanced (Figure 6B and C). These results indicated that OA/5FU combination therapy can affect cell apoptosis by regulating calcium channel inhibitors.

## Discussion

HCC is the most common primary liver cancer that originates from hepatocytes. Its incidence and mortality rates are both high, accounting for 85-90% of all liver cancer cases (22). HCC is usually associated with diverse chronic liver diseases, such as hepatitis B virus (HBV) infection, non-alcoholic fatty liver disease (NAFLD), and certain hereditary liver diseases (such as hemochromatosis) (23). The prognosis of HCC is poor, especially in late stages. Although surgical resection and local treatment can improve the survival rate of early-stage patients, long-term survival still faces challenges due to the high recurrence rate of HCC (24). Therefore, exploring new diagnostic methods and treatment strategies can provide hope for improving the prognosis of patients with HCC.

Research has demonstrated that OA, a terpenoid substance extensively present in plants, plays a clear role in alleviating obesity, alcoholic fatty liver, liver damage, and other conditions (25). OA is a pentacyclic triterpenoid compound used clinically for treating acute and chronic hepatitis. Liao et al. (26) found that OA inhibits SIRT1 protein expression. SIRT1 activation by the agonist SRT1720 significantly improves OA-induced liver toxicity. Calcium ions (Ca^2+^) are important secondary messengers that play crucial roles in intracellular signal transduction, cell proliferation, apoptosis, and metabolism (27). It is reported that OA is a calcium modulator, which is involved in progress of calcium-independent release of endothelium-derived nitric oxide and phospholipase C-calcium dependent signaling pathway. In addition, OA can also block secretory phospholipase A2 IIA-activated calcium signals (28-30). OA induces tumor cell apoptosis by regulating calcium ion channels and signaling pathways. OA can upregulate the intracellular calcium ion concentration and activate calcium-dependent enzymes, such as caspases. This triggers the apoptotic signaling pathway leading to the apoptosis of tumor cells. Herein, OA inhibited HCC cell proliferation in a concentration-dependent manner and promoted their ability to undergo apoptosis. Although the results indicated that OA has antitumor potential, further research is required to verify its safety.

Research has found that 5-fluorouridine triphosphate (FUTP) generated by 5FU metabolism can be incorporated into RNA, interfering with RNA synthesis and function and leading to the inhibition of cellular protein synthesis (31). Additionally, another metabolite of 5FU, 5-fluoro-2'-deoxyuridine triphosphate (FdUTP), can be incorporated into DNA, causing DNA damage and breakage, further triggering cell apoptosis (32). However, the efficacy of 5FU monotherapy in the treatment of tumors is limited. Owing to the heterogeneity of colorectal cancer and natural resistance to chemotherapy drugs, the response rate to 5FU alone is relatively low, but it is still used as a treatment option in some cases (33). Doi et al. (34) found that 5FU is often combined with cisplatin for use in transarterial chemoembolization (TACE) as part of local treatment. This method involves direct administration through the hepatic artery, which increases local drug concentration and reduces systemic toxicity. TACE+5FU treatment controls tumor growth locally and prolongs patient survival. In advanced liver cancer, a combination of 5FU and genistein (GEN) is used to improve treatment efficacy. This combination therapy may synergistically inhibit tumor progression through diverse mechanisms of action (35). This study is the first to explore the synergistic treatment of liver cancer with OA and 5FU, and to provide potential therapeutic targets for liver cancer patients in clinical practice.

Calcium ion channels (Ca^2+^) not only regulate cellular signal conduction, cell growth, and differentiation, but also play an important role in cancer development. In Ca^2+^ signaling pathway, Grp75, PDI, and VDAC1 jointly participate in regulating the calcium homeostasis between the endoplasmic reticulum and mitochondria. For example, the voltage-dependent anion channel VDAC1 acts as the main channel for Ca^2+^ signaling pathway crossing the outer mitochondrial membrane, by binding to the molecular chaperones Grp75 and PDI and regulating the transport of Ca^2+^ from ER to the mitochondria (36). Herein, the therapeutic effect of the combination of the two drugs was validated by adding the calcium ion inhibitor 2-APB. Li et al. (37) found that inhibiting calcium channels interferes with the entry of calcium ions into cells and reduces the activity of calcium-dependent cell proliferation signaling pathways, thereby inhibiting cancer cell proliferation. Intracellular calcium homeostasis is disrupted by the inhibition of calcium channels, which may activate the apoptotic pathway and induce cancer cell death (38). Calcium channel inhibitors have potential in cancer treatment. In the present study, with the addition of the calcium channel inhibitor 2-APB, OA/5FU co-treatment regulated the expressions of Grp75, PDI, and VDAC1, the Ca^2+^ channel-related proteins, in liver cancer cells. Current studies also show that Grp75 and VDAC1 regulate the release of Ca^2+^ in cervical cancer and ovarian cancer (39,40). However, the specific mechanism of action of the calcium channel inhibitor 2-APB in liver cancer is currently unclear, and more research is required to determine its effectiveness and safety.

This study also has limitations, such as a lack of clinical evidence. Currently, research on the combination of OA and 5FU for treating HCC predominantly focuses on cellular experiments, and clinical studies to detect its specific mechanisms in the human body are lacking. Therefore, we should strengthen clinical research to evaluate the effectiveness of OA combined with 5FU in the treatment of HCC and improve its therapeutic effect.

In conclusion, the combination therapy of OA and 5FU significantly inhibited the cell proliferation and plate clone formation ability of HCC cells. The combination of the two drugs significantly induced the ability of cells to undergo apoptosis. Simultaneously, it enhanced Ca^2+^ signaling intensity in HCC. Overall, OA/5FU combination therapy should be tested as a new strategy for treating patients with HCC in clinical practice.

## Supplementary Material

Click to view [pdf].
