# Supplementary figures and images for: Oleanolic acid enhanced the anticancer effect of fluorouracil by regulating Ca2+ levels in hepatocellular carcinoma cells
Source: Braz J Med Biol Res. 2025 Apr 14;58:e14590. doi: 10.1590/1414-431X2025e14590 (PMC11996166; doi:10.1590/1414-431X2025e14590)

**Figure S1.** Combination index (CI) of oleanolic acid (OA) and fluorouracil (5-FU).

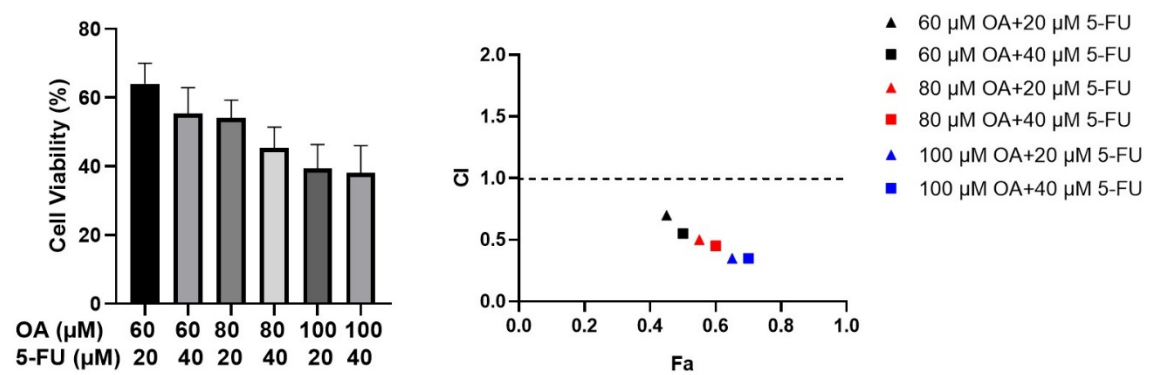

Supplement: Supplementary file 1 [file 1414-431X-bjmbr-58-e14590-suppl.pdf]
